# Traitement chirurgical des fractures du Calcanéum: à propos de 29 cas

**DOI:** 10.11604/pamj.2017.26.137.11462

**Published:** 2017-03-13

**Authors:** Badr El Alami, Aimane Naam, Mohamed Admi, Ilyas Rabhi, Mohamed Elbardai, Fawzi Boutayeb

**Affiliations:** 1 Service de Traumato-Orthopédie A, CHU Hassan II Fès, Maroc

**Keywords:** Fracture, calcanéum, chirurgie, Fracture, calcaneus, surgery

## Abstract

Les fractures du calcanéum sont peu fréquentes mais le plus souvent graves. Nous rapportons une série de 29 cas de fractures du calcanéum traités chirurgicalement dans le service de traumatologie orthopédie du CHU Hassan II de Fès. L'objectif de ce travail rétrospectif était de présenter les principes et d'évaluer les résultats du traitement chirurgical des fractures articulaires du calcanéum, en comparaison avec le traitement conservateur. Il s'agissait de 21 hommes et de 8 femmes, dont l'âge variait entre 21 et 61 ans. L'étiologie était dominée par les accidents de la voie publique et les chutes d'un lieu élevé. L'évaluation des lésions était basée sur la classification de DUPARC. Le traitement a consisté à faire une réduction à foyer ouvert et une ostéosynthèse par plaque en Y, ou par une plaque tiers de tube réalisant un montage en triangulation. Les résultats cliniques ont été évalués en se basant sur le score de kitaoka, avec un recul moyen de 24 mois. 86% de nos patients ont eu un bon à moyen résultat.

## Abstract (en)

Calcaneal fractures are infrequent but, more often, serious. We report a series of 29 cases of calcaneal fractures surgically treated in the Orthopaedics and Traumatology in the University Hospital (CHU) Hassan II of Fez. This retrospective study aims to present the principles and to evaluate the results of surgical treatment for articular fractures of the calcaneus, in comparison with conservative treatment. Our study included 21 men and 8 women, the average age was 21-61 years. Etiology was dominated by road traffic accidents as wel as by falls from a high place. The evaluation of the lesions was based on the classification of Duparc. The treatment was based on open reduction associated with Y-plate osteosynthesis or one-third tubular plate osteosynthesis using triangulation. Clinical results were evaluated on the basis of kitaoka score, with an average follow-up period of 24 months. 86% of patients have had good medium-term results.

## Introduction

Les fractures du calcanéum sont des lésions graves. Elles représentent 65% des traumatismes du tarse et 2% de toutes les fractures. Les fractures thalamiques constituent une entité particulière par leur mécanisme de survenue, par leur traitement et par leur pronostic beaucoup moins favorable que les fractures extra-articulaires. Elles relèvent souvent d´un traitement chirurgical qui vise à restaurer l´anatomie de l´articulation sous-talienne. Les auteurs rapportent dans ce travail, les résultats d´une série de 29 cas de fractures articulaires du calcanéum, traitées par un abord externe, une réduction, et une ostéosynthèse par une plaque anatomique ou plaque tiers de tube montées en triangulation, avec une révision clinique et radiologique jusqu´au dernier recul.

## Méthodes

Il s'agit d'une étude rétrospective continue d'une série de 29 fractures articulaires du calcanéum traitées et suivi dans le service de chirurgie orthopédique du CHU Hassan II de FES entre le 1^er^ janvier 2010 et le 1^er^ janvier 2016 avec un recul moyen de 24 mois. L'exploration radiologique été basée sur un cliché de cheville de face et de profil et une incidence rétro-tibiale, et un scanner de l'arrière pied. On a utilisé la classification de DUPARC [1] pour sa valeur pédagogique permettant de mieux comprendre l'anatomo-pathologie des fractures thalamiques du calcanéum. L'intervention était pratiquée sous anesthésie générale ou loco-régionale. Le patient était installé en décubitus latéral. Nous avons utilisé une voie d'abord latérale rétro et sous-malléolaire en L élargie. La dissection était faite sans décollement jusqu'au périoste. Par une moucheture cutanée, une broche de Steinman était placée transversalement dans la grosse tubérosité. La traction vers le bas sur cette broche permettait d'améliorer l'exposition de la surface thalamique, d'abaisser la grosse tubérosité et de corriger son varus. Le deuxième temps consistait à réduire la surface thalamique fixée par des broches provisoires et perpendiculaires au trait fondamental. Un contrôle par amplificateur de brillance était fait pour vérifier le rétablissement de la hauteur calcanéenne, la qualité du relèvement, la correction de la congruence de la surface thalamique et enfin s'en suit l'ostéosynthèse définitive par une plaque anatomique ou trois plaques tiers de tube montées en triangulation sur la face externe du calcanéum. On a procédé à une immobilisation de la cheville par une attelle plâtrée pendant 15 jours, relayée après ablation de fils par une botte en résine pendant 45 jours. La rééducation était systématique chez tous les patients et l'appui partiel a été recommandé à la 9ème semaine et l'appui définitif n'a été autorisé qu'après 3 mois. Nos résultats ont été évalués selon l'angle de Bohler post-opératoire et le score fonctionnel de kitaoka.

## Résultats

**Figure 1:**
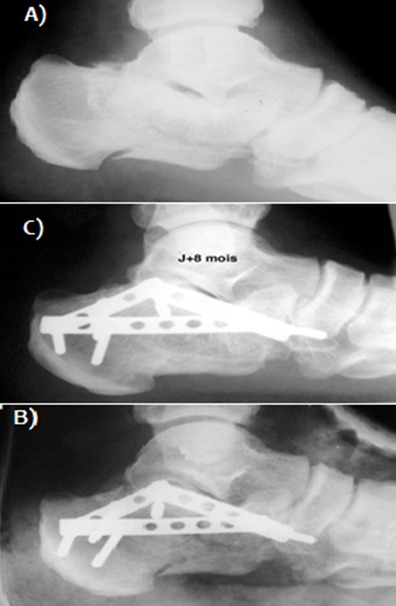
A) radiographie de cheville (profil) montrant une fracture du calcanéum stade III DUPARC; B) radiographie post opératoire-ostéosynthèse par plaque 1/3 de tube en triangulation; C) contrôle radiologique de la fracture à 8 mois

**Figure 2:**
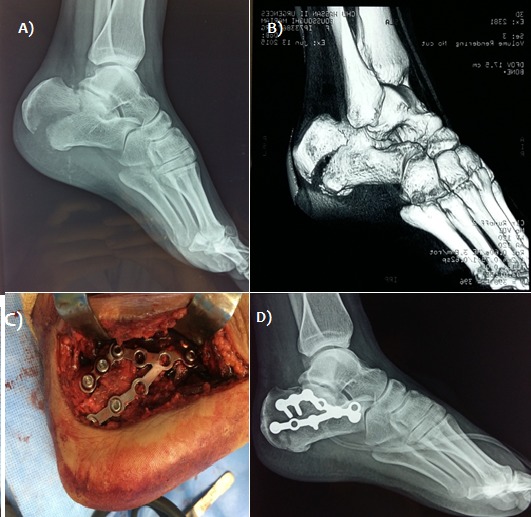
A) radiographie de cheville (profil) montrant une fracture du calcanéum stade III; B) reconstruction scannographique 3D de la fracture; C) image per-opératoire de la plaque anatomique du calcanéum; D) : radiographie post opératoire -ostéosynthèse par plaque anatomique

**Figure 3:**
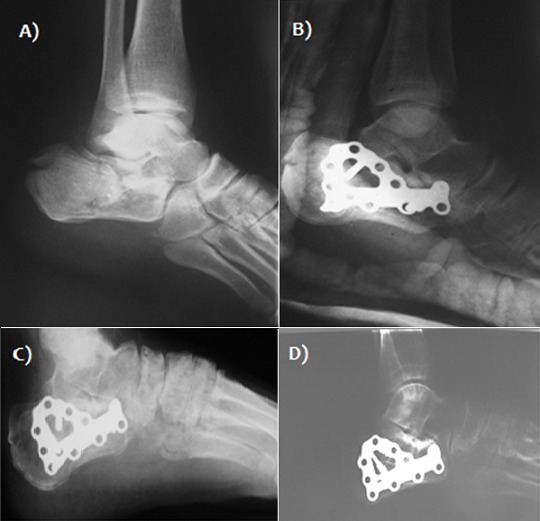
A) radiographie de cheville (profil) montrant une fracture du calcanéum stade IV; B) radiographie post opératoire-ostéosynthèse par une plaque anatomique en Y; C) contrôle radiologique de la fracture à 3 mois; D) contrôle radiologique de la fracture à un an

Il s'agissait de 21 hommes et 8 femmes, d'âge moyen 42 ans (extrêmes 21 à 61 ans). Le côté droit était atteint 20 fois, le côté gauche 9 fois. La fracture était bilatérale chez deux patients. Des lésions cutanées superficielles (phlyctènes) étaient présentes initialement dans 4 cas et deux cas ont présenté une ouverture cutanée médiale stade II de cauchois et Duparc. Les étiologies sont dominées par les accidents de la voie publique (22 cas soit 75%) et les chutes (7 cas soit 25%) avec 2 cas de défenestration volontaire en contexte psychiatrique (3 fractures). Des lésions associées de l'appareil locomoteur étaient observées chez six patients (2 fractures de fémur, une fracture du pilon tibial controlatéral, une fracture du talus homolatéral, deux fractures lombaires, une fracture sacrée, deux fractures de poignet). Selon la classification de DUPARC et de LA CAFFINIERE [1], Les fractures étaient types III de Duparc dans 48.2% des cas (14 patients) (Figure 1, Figure 2), types IV dans 37,9% (11 patients) (Figure 3) et type V dans 13,9% des cas (4 patients). L'enfoncement thalamique était de type vertical dans 8 cas, horizontal dans 6 cas et mixte dans 10 cas. L'ostéosynthèse été pratiquée entre le 4 ^ème^ et le 7 ^ème^ jour du post-traumatisme par une plaque anatomique (Figure 2, Figure 3) chez 18 patients (62%) et par 2 ou 3 plaques 1/3 de tube (Figure 1) chez 7 patients réalisant un montage en triangulation selon la direction des travées osseuses principales (24%) et vissage pour 4 patients (14%). Nous avons eu recours à une greffe cortico-spongieuse de soutien chez 4 patients (14%). Il s'agissait de 21 hommes et 8 femmes, d'âge moyen 42 ans (extrêmes 21 à 61 ans). Le côté droit était atteint 20 fois, le côté gauche 9 fois. La fracture était bilatérale chez deux patients. Des lésions cutanées superficielles (phlyctènes) étaient présentes initialement dans 4 cas et deux cas ont présenté une ouverture cutanée médiale stade II de cauchois et Duparc. Les étiologies sont dominées par les accidents de la voie publique (22 cas soit 75%) et les chutes (7 cas soit 25%) avec 2 cas de défenestration volontaire en contexte psychiatrique (3 fractures). Des lésions associées de l'appareil locomoteur étaient observées chez six patients (2 fractures de fémur, une fracture du pilon tibial controlatéral, une fracture du talus homolatéral, deux fractures lombaires, une fracture sacrée, deux fractures de poignet). Selon la classification de DUPARC et de LA CAFFINIERE [1], Les fractures étaient types III de Duparc dans 48.2% des cas (14 patients) (Figure 1, Figure 2), types IV dans 37,9% (11 patients) (Figure 3), et type V dans 13,9% des cas (4 patients). L'enfoncement thalamique était de type vertical dans 8 cas, horizontal dans 6 cas et mixte dans 10 cas. L'ostéosynthèse été pratiquée entre le 4 ^ème^ et le 7 ^ème^ jour du post-traumatisme par une plaque anatomique (Figure 2, Figure 3) chez 18 patients (62%) et par 2 ou 3 plaques 1/3 de tube (Figure 1) chez 7 patients réalisant un montage en triangulation selon la direction des travées osseuses principales (24%) et vissage pour 4 patients (14% ). Nous avons eu recours à une greffe cortico-spongieuse de soutien chez 4 patients (14%).

**Méthodes de révision:** Tous les patients ont été revus avec un recul moyen de 2 ans (12-60 mois). Les résultats fonctionnels ont été analysés selon la cotation de KITAOKA et al. [2] qui tient compte de 3 paramètres : la douleur, la fonction et l'alignement de l'arrière pied. Ainsi, le résultat a été considéré excellent, lorsque le score global était entre 95 et 100, bon lorsqu'il était entre 80 et 94, moyen lorsqu'il était entre 50 et 79 et mauvais lorsqu'il était inférieur à 50. L'évaluation des résultats anatomiques a été faite sur des radios de la cheville de face et de profil en charge et une incidence rétro-tibiale ascendante. Nous avons retenu la cotation de BABIN et al. [3] basée sur la mesure de l'angle de BÖHLER et le résultat anatomique était considéré très bon lorsque l'angle de BÖHLER était supérieur ou égal à 25°, bon quand il était compris entre 20° et 25°, passable quand il était compris entre 10° et 20° et mauvais quand il était inférieur à 10°.

**Résultats fonctionnels:** Selon la cotation de Kitaoka [2], le score moyen était de 74. Les résultats fonctionnels étaient excellent dans 16% des cas, bon dans 56% des cas, moyen dans 25% des cas et mauvais dans 3% des cas (repris par une double arthrodèse). La reprise du travail s'est faite en moyenne 4.6 mois avec les mêmes capacités chez 10 patients (34%), deux patients avaient une nette diminution physique. L'axe de l'arrière pied était normal pour 83% et un pied plat valgus a été retrouvé dans 17% des cas.

**Résultats radiologiques:** Une arthrose talo-calcanéenne (Figure 3) était apparue dans 4 cas au dernier recul, incluant 3 cas de vissage et dont un cas a été repris par arthrodèse sous-talienne. Une arthrose calcanéo-cuboidienne était apparue dans 3 cas et 2 cas ont présenté une arthrose tibio-talienne. Sur le plan anatomique, le résultat était très bon dans 24% des cas, bon dans 8% des cas, moyen dans 28% des cas et mauvais dans 40% des cas. L'angle de Böhler moyen au dernier recul était de 13° (0°à 35°). L'étude de la différence entre l'angle de Böhler calculé en postopératoire immédiat et au dernier recul montrait une perte secondaire du relèvement initial de la surface thalamique qui était en moyenne de 3° (0°à 5°). Ce tassement secondaire n'a intéressé que 48% des patients.

**Complications:** Nous avons noté un cas de retard de cicatrisation cutanée et un cas d'infection superficielle qui ont bien évolué sous antibiothérapie et soins locaux. Un cas de nécrose cutanée a bénéficié d´une greffe cutanée. Quatre patients ont développé une algodystrophie qui a favorablement évolué sous traitement médical et rééducation. Enfin, au dernier recul, nous avons noté un cas d'arthrose sous-talienne mal tolérée qui a nécessité une double arthrodèse.

## Discussion

Depuis les premières descriptions de ces fractures thalamiques, leur traitement reste controversé [4, 5]. En 1931, Böhler [6] décrivait la méthode de réduction par une broche de traction suivie d'une immobilisation par plâtre. En 1913, Leriche recommandait une ostéosynthèse par plaque et vis [7]. Ces recommandations ont été appuyées par Palmer [8]. Ces dernières années, grâce à une meilleure analyse des lésions anatomo-pathologiques et à l'établissement de bases techniques de la réduction et de l'ostéosynthèse à ciel ouvert des fractures articulaires du calcanéum, beaucoup d'auteurs ont rapporté des résultats satisfaisants après le traitement chirurgical : Buckley et Meek [5], Crosby et Fitzgibbons [9] et Parmar [10]. Ce traitement fait partie actuellement de l'arsenal thérapeutique des fractures articulaires du calcanéum. L'analyse des séries comparatives, traitement chirurgical versus traitement fonctionnel des fractures articulaires du calcanéum a montré que le traitement chirurgical permet d'aboutir à des résultats comparables, voir supérieurs au traitement fonctionnel [11, 12]. Parma et al. [10] ont publié en 1993 la première étude comparative randomisée de 31 patients traités fonctionnellement et 25 patients traités chirurgicalement. Avec un recul moyen de 23 mois, les résultats fonctionnels étaient excellents et bons chez 65% des patients non opérés et chez 64% des patients opérés. Buckley et Meek [5] ont publié les résultats d'une étude comparative randomisée de 36 patients dont 17 traités fonctionnellement et 19 traités chirurgicalement. Avec un recul moyen de 5 ans, les résultats fonctionnels étaient bons et très bons dans 69% des cas pour le premier groupe et dans 65% des cas pour le second. Les auteurs constataient cependant que les patients parfaitement réduits chirurgicalement présentaient un meilleur résultat que les autres et concluaient à la supériorité du traitement chirurgical si l'on est certain de pouvoir obtenir une réduction parfaite. Crosby et Fitzgibbons [9] ont obtenu 100% de bons et très bons résultats pour le groupe opéré et 20% pour le groupe non opéré. Pour ces auteurs, devant toute fracture déplacée, le traitement chirurgical s'impose. Ainsi, nous rejoignons la plupart des auteurs pour dire que le traitement des fractures articulaires déplacées du calcanéum doit être chirurgical. Le risque de nécrose cutanée et d'infection, inconvénients majeurs de ce traitement, peut être nettement diminué par certaines règles : avant l'intervention, il faut veiller à diminuer l'œdème et les phénomènes inflammatoires par la surélévation du membre, le glaçage et l'administration d'un traitement anti-inflammatoire. L'intervention doit être décidée dès la résolution des phénomènes inflammatoires aux alentours du 7 ^ème^ jour, l'incision cutanée doit être latéralisée à proximité du tendon d'Achille. Il faut éviter les angles aigus, la dissection doit être limitée et le lambeau supérieur doit être soulevé à partir du périoste emportant les tendons fibulaires et le nerf sural. La fermeture de l'incision doit se faire sans tension en deux plans sous drainage. En postopératoire, le membre doit être maintenu surélevé pendant quelques jours. Il faut éviter les pansements compressifs. L'ablation des fils doit se faire à la 3e semaine. En respectant ces règles, nos complications cutanées et septiques ont été rares et sans incidence grave sur le résultat final, par rapport à la série de Stephenson où le taux de nécrose cutanée été de l'ordre de 21% [13].

La technique d'ostéosynthèse ne fait pas l'unanimité des auteurs. L'ostéosynthèse par plaque et le vissage constituent les principaux moyens d'ostéosynthèse. Schmidt et al. [14], dans une étude multicentrique réalisée par la Société française de chirurgie orthopédique et traumatologique portant sur 1 071 cas, ont trouvé 54% de résultats fonctionnels satisfaisants après une ostéosynthèse conventionnelle (vissage, brochage,…) et 71% de résultats satisfaisants après une ostéosynthèse par plaque. Bezes et al. [15], sur une série de 20 patients traités par une ostéosynthèse par plaque, ont rapporté 85% de résultats satisfaisants. Nous n'avons pas retrouvé dans la littérature de séries comparatives d'ostéosynthèse par vissage versus ostéosynthèse par plaque. Globalement, les résultats des ostéosynthèses par vissage sont satisfaisants dans 71 à 77% dans la série de Chaminade et al. [16] et de Stephenson [13], donc les résultats des ostéosynthèses par plaque ne paraissent pas sensiblement supérieurs aux résultats du vissage. Dans notre série, la perte de correction secondaire observée n'était que de 3° en moyenne. D'ailleurs, cette valeur est comparable aux pertes secondaires après une ostéosynthèse par plaque publiées par Thermann et al. [17]. Enfin, la technique de vissage expose à un risque mineur de complications cutanées et infectieuses contrairement aux ostéosynthèses par plaque où ce risque peut atteindre 30% surtout lorsqu'il s'agit de plaques non adaptées comme cela a été souligné par Levin et Nunley [18]. L'adjonction d'une greffe osseuse demeure un sujet de discussion. Wilmoth [19] fut le premier à proposer l'adjonction d'une greffe osseuse pour combler le vide créé par le relèvement de la surface thalamique. Palmer [8] a appuyé cette idée dans le but supplémentaire de renforcer la stabilité du relèvement. Depuis ces descriptions, certains auteurs ont utilisé systématiquement une greffe osseuse. Cette attitude n'a pas été partagée par Geel et Flemister [20] et Letournel [21]. Ce dernier pense que la greffe osseuse n'est pas nécessaire parce que les vis sont capables à elles seules de stabiliser le relèvement de la surface thalamique. Stephenson [16] a rapporté, dans Son étude, qu'il n'a jamais utilisé de greffe osseuse et il n'a noté qu'un seul cas de tassement secondaire. Sanders et al. [22], dans une série de 120 cas traités chirurgicalement sans greffe osseuse, n'ont pas noté de déplacement secondaire. Dans notre série, la greffe osseuse été jugé nécessaire dans 14% des cas face à des défects osseux importants. L´arthrose sous talienne constitue la complication la plus redoutable au long court. Elle est estimée à 5,6% dans la série de Zwipp comportant 194 patients [23]. Ce risque semble diminué dans les suites d´une ostéosynthèse par plaque [23], dans notre série cette complication a été révélée chez 13% des cas dont un a nécessité une double arthrodèse de l'arrière pied. Nos résultats fonctionnels étaient comparables aux données de la littérature avec 72% de bon à très bon résultats.

## Conclusion

Le traitement chirurgical des fractures articulaires déplacées du calcanéum est recommandé par la plupart des auteurs contemporains. Les publications récentes ont montré une amélioration des résultats fonctionnel et radiologique après réduction chirurgicale et ostéosynthèse, en comparaison avec le traitement conservateur. Les principes répondent aux objectifs du traitement des fractures articulaires appliquées aux particularités anatomiques de l'os calcanéen. Il s'agit de rétablir l'anatomie et la surface articulaire thalamique, d'obtenir un montage stable et de limiter les complications (cutané, arthrose sous-talienne). Conflits d'intérêts

### Etat des connaissances actuelles sur le sujet

- Les fractures articulaires déplacées du calcanéum relèvent d'un traitement chirurgical et leur moyen d'ostéosynthèse ne fait pas l'unanimité des auteurs;
- La voie d'abord externe est la plus recommandée malgré le risque élevé de nécrose cutanée.

### Contribution de notre étude à la connaissance

- L'ostéosynthèse par plaque semble être meilleure en matière de réduction, du rétablissement durable de l'angle de Bohler ce qui réduit le taux d'arthrose sous-talienne;
- Dans notre série nous avons objectivé un taux de 4% d'arthrose sous talienne pour les patients qui ont bénéficié d'une ostéosynthèse par plaque alors que les séries de la littérature rapporte un taux minimal de 5,6%;
- L'incision cutanée courbée au niveau de l'angle semble éviter la nécrose cutanée, complication redoutable de la voie d'abord externe. Notre série rapporte un taux de 3,4% de nécrose cutanée alors que les séries de la littérature rapporte des taux qui peuvent atteindre 30% dans la série de LEVIN.
